# Mechano-fluorochromic behavior of AEE polyurethane films and their high sensitivity to halogen acid gas†

**DOI:** 10.1039/c8ra10486g

**Published:** 2019-03-26

**Authors:** Kun Wang, Meng Wang, Hao Lu, Beibei Liu, Mingming Huang, Jiping Yang

**Affiliations:** Key Laboratory of Aerospace Advanced Materials and Performance, Ministry of Education, School of Materials Science and Engineering, Beihang University Beijing 100191 China jyang@buaa.edu.cn

## Abstract

Three polyurethanes with different contents of tetraaryl-buta-1,3-diene derivatives in the soft segment (STMPU-25/STMPU-50/STMPU-75) have been synthesized and found to present aggregation-enhanced emission features. The fluorescence intensity of polymer films was greatly enhanced with increasing tensile stress. Also, polyurethanes with higher aggregation-induced emission fluorogen content had stronger mechano-fluorochromic behavior in the same tension state. Moreover, the resulting polyurethane films possessed high sensitivity for halogen acid gas, suggesting their potential applications in environmental monitoring fields.

## Abstract 2

(a) Polyurethanes with higher content of TABDAA2 presented better positive correlation between fluorescence and strain. (b) Sensing mechanism of STMPU-50 for detection of HCl.

## Graphical abstract

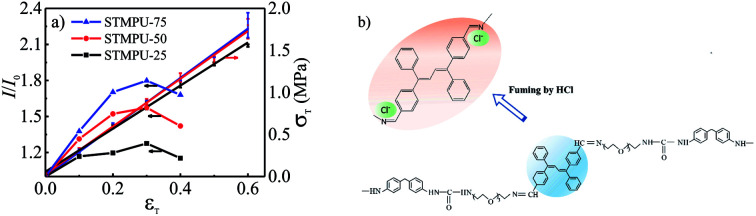

In contrast to traditional fluorescent compounds with the property of aggregation-caused quenching (ACQ), aggregation-induced emission (AIE) or aggregation-enhanced emission (AEE) molecules show strong emission in aggregation states, and do not emit fluorescence in solution.^1–3^ These AIE small molecules have been found in many luminescence systems such as silole,^1^ tetraphenylethene,^4,5^ styrylbenzene^6^ and their derivatives.^7^

Interestingly, many AIE compounds have been proved to be piezo- or mechano-fluorochromic materials.^8^ In 2008, Tang *et al.* found that the photoluminescence of hexaphenylsilole was enhanced by pressure in the solid state.^9^ In 2010, Park *et al.* reported a cyano-substituted distyryl benzene derivative, which showed reversible multi-stimuli luminescence switching.^10^ At the same time, Chi *et al.* firstly put forward the concept of piezofluorochromic aggregation-induced emission (PAIE) materials. They synthesized several PAIE materials and proposed that AIE compounds containing a twisted conformation would exhibit piezofluorochromic activity.^11^ Till now, numerous reported AIE compounds, such as typical triphenylethylene,^12^ tetraphenylethylene (TPE),^13–17^ silole,^18^ cyano-distyrylbenzene,^19–21^ 9,10-distyrylanthracene and their derivatives^22–31^ were found to possess PAIE feature.

However, AIE/AEE polymers possessing mechano-fluorochromic feature were rarely studied.^32–34^ Kokado *et al.* found that the AIE elastomer based on PDMS and TPE exhibited stimuli-sensitive fluorescence against organic solvents and temperature.^35^ Tang *et al.* reported a TPE-containing memory chromic polyurethane, which showed that the emission intensity of resulting polyurethane gave negative correlation with shape fixity, temperature, and existence of solvent.^36^ In our previous work, an AEE polyurethane containing 4,4′-((1*Z*,3*Z*)-1,4-diphenylbuta-1,3-diene-1,4-diyl) dibenzaldehyde (TABDAA2) was prepared and it was found that the fluorescence of polyurethane films was enhanced in the tension state.^37,38^ So far, the effect of tension on the fluorescent intensity of polymer films was poorly understood.

Meanwhile, the detection of halide is important for monitoring excessive halide levels in the environment (*i.e.* pollution in air and water). There are many ways to determine the concentration of the halide in the solutions, such as amperometry and chemistry titration,^39–42^ but few researches are about detecting the presence of halide gas in air.

Considering that the content of AIE molecules TABDAA2 might affect the fluorescence behavior of polyurethane films, three polyurethanes with different mass fraction (0.25%, 0.5% and 0.75%) of TABDAA2 in the soft segments (STMPU-25/STMPU-50/STMPU-75) have been synthesized in this work. And their mechano-fluorochromic behavior were investigated. Meanwhile, the polyurethane film was sensitive to halogen acid gas like hydrogen iodide (HI), proving that it could be good solid probe to halogen acid gas.

All the materials, instruments and synthesis procedure for STMPUs polyurethanes with modified reactant molar ratios have been shown in the previous articles and the ESI.†^37,38^ In brief, the mixture of TABDAA2 (0.25%, 0.5% and 0.75% mass fraction) and poly(tetrahydrofuran) (*M*_n_ = 1000, PTMG1000) was used as soft segments, and reacted with 4,4-diphenylmethane diisocyanate (MDI) and 1,4-butanediol (BDO) in the molar ratio of 1 : 2 : 1 to give the product polyurethanes STMPUs (STMPU-25/STMPU-50/STMPU-75). The reaction molar ratio, average molecular weight (*M*_n_) and polymer dispersion index (PDI) for STMPUs were listed in Table 1.

**Table 1:** Reactant molar ratios, *M*_n_ and PDI for STMPU-25, STMPU-50 and STMPU-75

STMPUs	MDI (mM)	PTMG (mM)	TABDAA2 (mM)	BDO (mM)	*M*_n_ (kDa)/PDI
STMPU-25	20.2	9.95	0.05	10.0	103/1.56
STMPU-50	20.2	9.90	0.10	10.0	148/1.43
STMPU-75	20.2	9.85	0.15	10.0	102/1.50

Firstly, the fluorescent emission spectra of STMPU-50 in the solution and aggregate states was investigated as shown in Fig. 1(a) and (b). The fluorescent intensity of STMPU-50 increased continuously until water fraction raised up to 80%. When the water fraction reached beyond 80%, the fluorescent emission started to decrease. These results demonstrated that resulting polyurethane indeed had an AEE feature. The fluorescent emission spectra of STMPU-25 and STMPU-75 in the solution and aggregate states were also studied (Fig. S1†). It can be found that STMPU-75 had the highest fluorescent intensity. And all three polyurethanes possessed AEE property. Dong *et al.* reported that tetraaryl-buta-1,3-diene (TABD) small molecules had typical AIE effect.^43,44^ When the moiety TABDAA2 was introduced into polyurethane systems, these polymers usually exhibited AEE feature rather than the typical AIE effect. Since the AIE small molecules were chemically located within the polymer matrix, which limited their intramolecular rotations to some extent. So, it could block the nonradioactive pathway and populate the radiative excitons, leading to the luminescence of the polyurethane in the solution state.

**Fig. 1:**
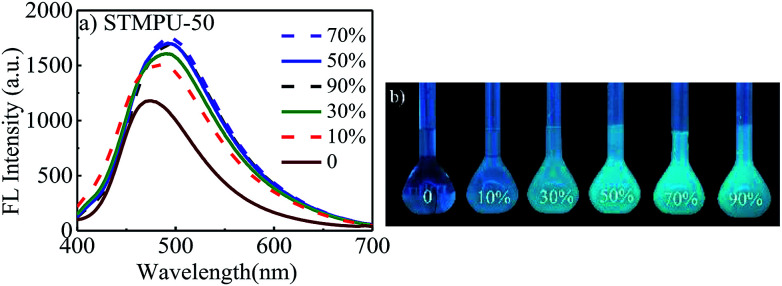
(a) Fluorescence emission spectra of STMPU-50 in DMF–water mixtures with different water fractions (polyurethane solution concentration: 10 mg mL^−1^; excitation wavelength: 365 nm); (b) photographs of polyurethane STMPU-50 solutions with different water fraction under 365 nm UV illumination.

To further investigate the mechano-fluorochromic properties of AEE polyurethanes, three polyurethanes with different mass fraction of TABDAA2 (0.25%, 0.50% and 0.75%) were synthesized and their FL-true strain spectra were depicted in Fig. 2(a)–(c). The fluorescent intensity of all STMPUs firstly increased and then decreased with the increased true strain. And their true stress–strain curves were conducted by tension experiment at room temperature (Fig. 2(d)), which exhibited typical elastomeric feature. The true strain (*ε*_T_)-dependent fluorescence spectra of three STMPUs were also summarized in Fig. 2(d). In the low strain scope (*ε*_T_ ≤ 0.3), the fluorescence intensity of the film was increased, while the stress was also increased with increased strain in the linear correlation coefficients (*ε*_T_ ≤ 0.3) of 0.9180, 0.8729 and 0.8608 for STMPU-25, STMPU-50 and STMPU-75, respectively, proving that polyurethanes with higher concentration of TABDAA2 had more positive correlation with the tensile strain when *ε*_T_ ≤ 0.3.

**Fig. 2:**
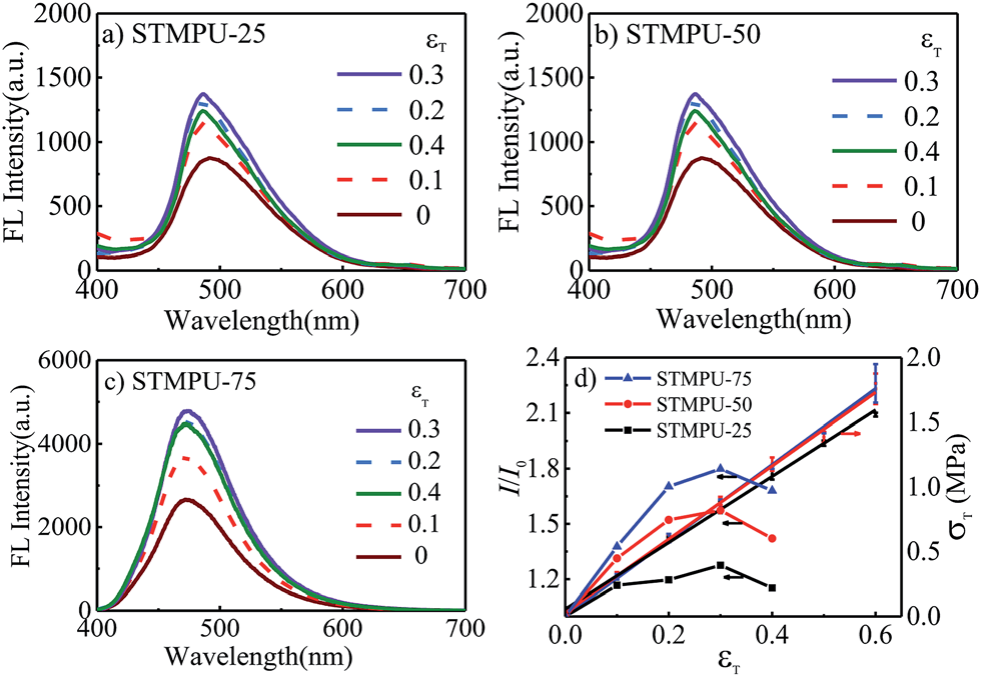
Fluorescence emission spectra of (a) STMPU-25, (b) STMPU-50 and (c) STMPU-75 films at different tensile strains; (d) true stress–strain curves and fluorescence peak intensity-true strain curves of three AEE polyurethanes.

XRD profiles of the STMPU-50 film before and after stretching were measured (Fig. S2†). After stretching, peaks at about 21.5° and 29° became sharper a little than the original state, suggesting that stretching can induce the more regular structure in the polyurethane film. Thus, we could assume that the tension stress could bring two opposite effects on the FL emission intensity of the STMPU films. In the initial stage of stretching, the structure of the polyurethane films became more regular and the aggregation state of AIE moiety in the soft segments of the polyurethanes was enhanced, thus leading to a rising fluorescence intensity. At the same time, the concentration of the TABDAA2 in the films dropped due to the enlarged volume of the film accompanied with the stretching, causing the reduction of the fluorescence intensity when the strain was further increased.^45^

As a result, polyurethanes with higher concentration of TABDAA2 might prevent some negative effect of stretching on fluorescence intensity, implying that high mass fraction of AIE moiety in the polyurethanes would lead to the more positive mechano-fluorochromic feature in a larger scope of strain. This novel positive mechano-fluorochromic feature has the potential to be applied widely in strain or stress sensors.

Meanwhile, it was observed that the fluorescent intensity of polyurethane films was very sensitive to hydrogen iodide. 1 mL halogen acid and 1 mL ammonia water (25–28% wt) was put into 5 mL beakers to prepare halogen acid gas and ammonia atmosphere. As seen in Fig. 3(a), when the STMPU-50 film was fumed with hydroiodic acid (HI, 45% wt) for 5 seconds, the fluorescence of the film was almost quenched. And after fumigating the film with ammonia, the FL intensity increased and surpassed the original state of the film. The bright and dark states could be inter-converted to each other 3 times in the fluorescent emission intensity (Fig. 3(b)), and these chemical stimuli were non-destructive.

**Fig. 3:**
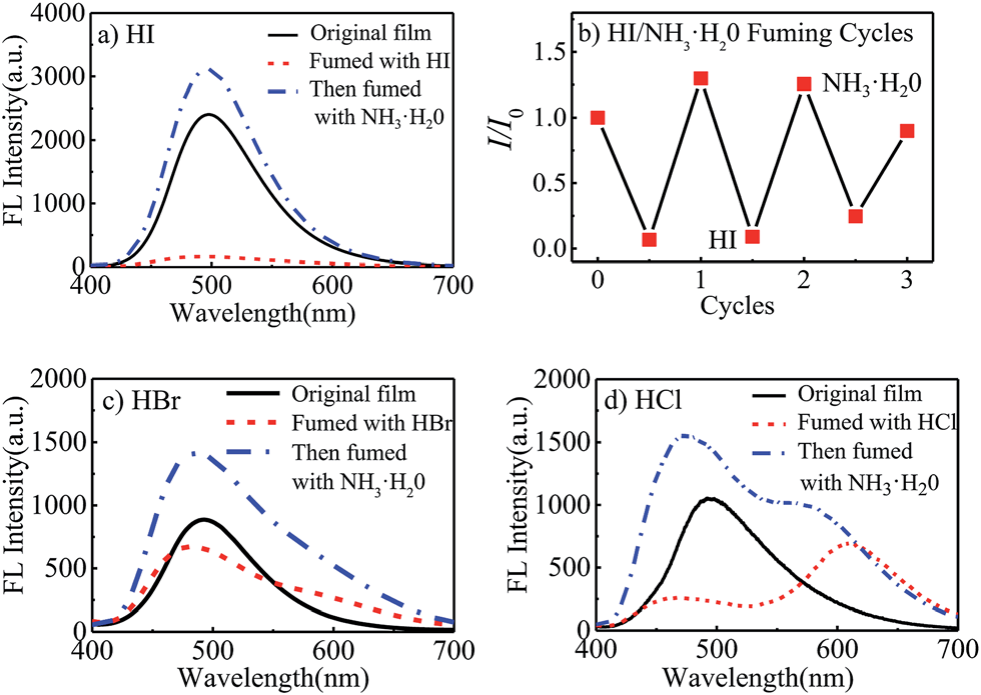
(a) Fluorescence emission spectra of STMPU-50 film before and after fumed with hydroiodic acid and then fumed with NH_3_·H_2_O; (b) the quenching and recovery test by HI–NH_3_·H_2_O fuming cycles; fluorescence emission spectra of STMPU-50 film before and after fumed with (c) hydrobromic acid and (d) hydrochloric acid, and then fumed with NH_3_·H_2_O.

When hydrobromic acid (HBr, 47% wt) was used to fume the polyurethane film, a new small shoulder peak was shown in Fig. 3(c). While after fuming the film with ammonia, the shoulder peak was disappeared, and the FL intensity was enhanced comparing to the original state of the film.

Further, Fig. 3(d) showed that the STMPU-50 film was fumigated with hydrochloric acid (HCl, 37% wt), an obvious new emission peak at around 610 nm appeared, but the original peak at 480 nm disappeared mostly. When the film was smoked with ammonia, the original fluorescent emission peak was enhanced, but the new emission peak in 610 nm became shoulder peak. Therefore, it could be assumed that the polyurethane film had different and obvious response to hydrogen iodide and hydrochloric acid.

Simultaneously, this phenomenon could be observed visually in Fig. 4(a) and (b). The STMPU-50 film was deep yellow after smoked with hydroiodic acid, then turned to pale yellow when fumed with NH_3_·H_2_O. Under 365 nm ultraviolet lamp, the original film exhibited blue fluorescence, then got dim greenish blue after fumigated with hydroiodic acid. The fluorescence of film became bright blue when fumigated with NH_3_·H_2_O. In contrast, the film was painted reddish brown after smoked with hydrochloric acid, and then turned to pale yellow when fumed with NH_3_·H_2_O. Under 365 nm ultraviolet lamp, the original film exhibited blue fluorescence, and yellow after smoked with hydrochloric acid. The fluorescence of film became green when fumigated with NH_3_·H_2_O.

**Fig. 4:**
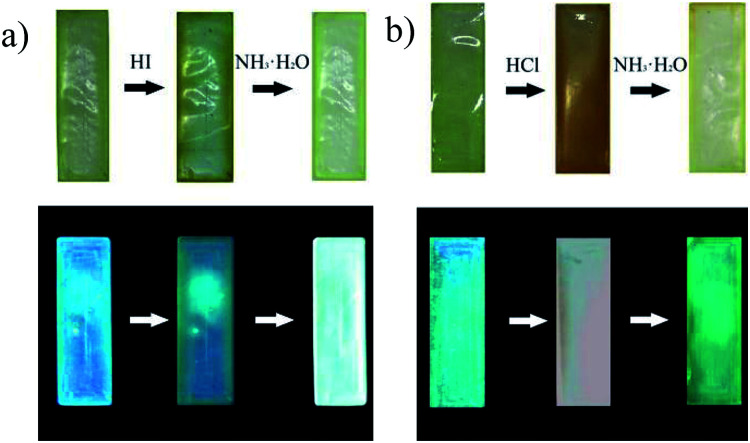
(a) Color changes of the STMPU-50 film before and after fumed with hydroiodic acid and then smoked with NH_3_·H_2_O under normal light (top) and fluorescent changes upon irradiation by 365 nm ultraviolet lamp (bottom); (b) color changes of the STMPU-50 film before and after fumigated with hydrochloric acid and then fumed with NH_3_·H_2_O under normal light (top) and fluorescence changes upon irradiation by 365 nm ultraviolet lamp (bottom).

According to the fluorescent color and brightness changes of the STMPU-50 film, the proposed sensing mechanism was illustrated in Fig. 5. Most of the Schiff base moiety of STMPU-50 with Cl^−^ formed a highly rigid fluorophore so that the conjugation extent of the molecular chain was enlarged, leading to a red shift peak at 610 nm. And for HBr, part of TABDAA2 molecules could form a rigid ring with the Schiff base moiety so that the new peak in 610 nm was weak as a shoulder peak. However, I^−^ ion was too large to insert into the space between C=N and the benzene ring of the TABD.^46,47^ Thus, I^−^ ion reacted with TABD fluorogen by electrostatic interactions and offered more diffusion channels for the excitons to migrate, allowing them to be more quickly annihilated by the hydrogen iodide.^48^ Next, when these films were fumed with ammonia gas, halogen ion could react with NH_4_^+^ due to the acid–base neutralization. Thus, the emission peak at around 470 nm was enhanced again.

**Fig. 5:**
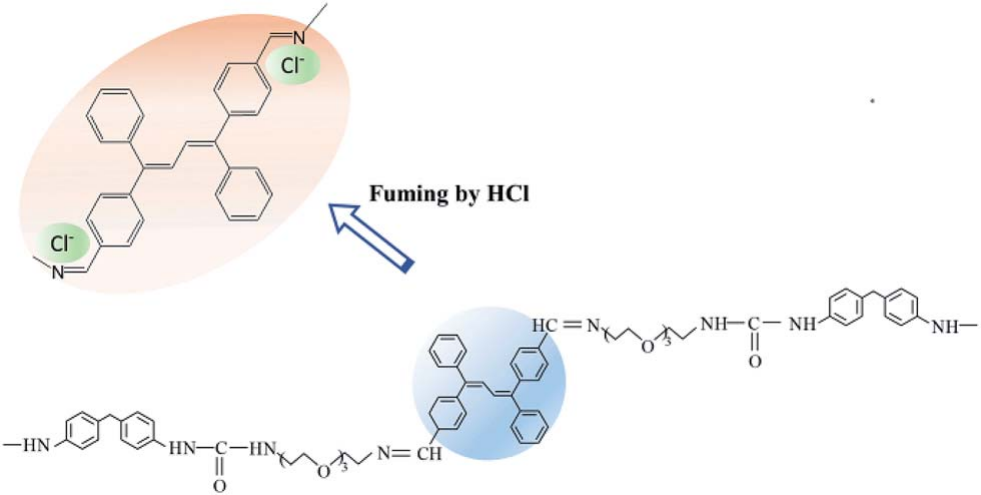
The proposed sensing mechanism of the STMPU-50 for the detection of HCl gas.

In summary, three aggregation-enhanced emission polyurethanes with 0.25%, 0.5% and 0.75% of tetraaryl-buta-1,3-diene derivatives in the soft segments, that is, STMPU-25/STMPU-50/STMPU-75, have been synthesized. The fluorescence intensity of the polymer films was greatly increased with the increase of tensile stress. Also, polyurethanes with higher content of AIE fluorogen presented better positive correlation between fluorescent intensity and tensile strain when *ε*_T_ ≤ 0.3. Moreover, the resulting polyurethane films had high sensitivity for halogen acid gas, suggesting their potential applications in environment monitoring fields.

## Conflicts of interest

There are no conflicts to declare.

## Supplementary Material

RA-009-C8RA10486G-s001

## References

[cit1] Luo J., Xie Z., Lam J., Cheng L., Chen H., Qiu C., Kwok H., Zhan X., Liu Y., Zhu D. (2001). Chem. Commun..

[cit2] Hong Y., Lam J., Tang B. (2011). Chem. Soc. Rev..

[cit3] Mei J., Hong Y., Lam J., Qin A., Tang Y., Tang B. (2014). Adv. Mater..

[cit4] Dong Y., Lam J., Qin A., Liu J., Li Z., Tang B., Sun J., Kwok H. (2007). Appl. Phys. Lett..

[cit5] Samuel A. C., Gil S., Costero A. M. (2017). RSC Adv..

[cit6] Xie Z., Yang B., Xie W., Liu L., Shen F., Wang H., Yang X., Wang Z., Li Y., Hanif M. (2006). J. Phys. Chem. B.

[cit7] Zeng Q., Li Z., Dong Y., Qin A., Hong Y., Ji L., Zhu Z., Jim C., Yu G., Li Q. (2007). Chem. Commun..

[cit8] Chi Z., Zhang X., Xu B., Zhou X., Ma C., Zhang Y., Liu S., Xu J. (2012). Chem. Soc. Rev..

[cit9] Fan X., Sun J., Wang F., Chu Z., Wang P., Dong Y., Hu R., Tang B., Zou D. (2008). Chem. Commun..

[cit10] Yoon S., Chung J., Gierschner J., Kim K., Choi M., Kim D., Park S. (2010). J. Am. Chem. Soc..

[cit11] Zhang X., Chi Z., Li H., Xu B., Li X., Zhou W., Liu S., Zhang Y., Xu J. (2011). Chem.–Asian J..

[cit12] Xu B., Chi Z., Zhang J., Zhang X., Li H., Li X., Liu S., Zhang Y., Xu J. (2011). Chem.–Asian J..

[cit13] Shi J., Chang N., Li C., Mei J., Deng C., Luo X., Liu Z., Bo Z., Dong Y., Tang B. (2012). Chem. Commun..

[cit14] Wang J., Mei J., Hu R., Sun J., Qin A., Tang B. (2012). J. Am. Chem. Soc..

[cit15] Zhou X., Li H., Chi Z., Zhang X., Zhang J., Xu B., Zhang Y., Liu S., Xu J. (2012). New J. Chem..

[cit16] Qi Q., Liu Y., Fang X., Zhang Y., Chen P., Wang Y., Yang B., Xu B., Tian W., Zhang S. (2013). RSC Adv..

[cit17] Cui Y., Yin Y., Cao H., Zhang M., Shan G., Sun H., Wu Y., Su Z., Xie W. (2015). Dyes Pigm..

[cit18] Mei J., Wang J., Qin A., Zhao H., Yuan W., Zhao Z., Sung H., Deng C., Zhang S., Williams I. (2012). J. Mater. Chem..

[cit19] Qin A., Zhang Y., Han N., Mei J., Sun J., Fan W., Tang B. (2012). Sci. China: Chem..

[cit20] Hou X., Ling J., Arulsamy N., Huo J. (2013). Mater. Sci. Appl..

[cit21] Ouyang M., Zhan L., Lv X. J., Cao F., Li W., Zhang Y. J., Wang K., Zhang C. (2015). RSC Adv..

[cit22] Zhang X., Chi Z., Zhang J., Li H., Xu B., Li X., Liu S., Zhang Y., Xu J. (2011). J. Phys. Chem. B.

[cit23] Bu L., Li Y., Wang J., Sun M., Zheng M., Liu W., Xue S., Yang W. (2013). Dyes Pigm..

[cit24] Bu L., Sun M., Zhang D., Liu W., Wang Y., Zheng M., Xue S., Yang W. (2013). J. Mater. Chem. C.

[cit25] Rao R., Liao C., Sun S. (2013). J. Mater. Chem. C.

[cit26] Yen H., Chen C., Liou G. (2013). Chem. Commun..

[cit27] He B., Chang Z., Jiang Y., Xu X., Lu P., Kwok H., Zhou J., Qiu H., Zhao Z., Tang B. (2014). Dyes Pigm..

[cit28] Li R., Xiao S., Li Y., Lin Q., Zhang R., Zhao J., Yang C., Zou K., Li D., Yi T. (2014). Chem. Sci..

[cit29] Sun M., Zhang D., Li Y., Wang J., Gao Y., Yang W. (2014). J. Lumin..

[cit30] Zheng M., Zhang D., Sun M., Li Y., Liu T., Xue S., Yang W. (2014). J. Mater. Chem. C.

[cit31] Yang M., Zhang Y., Zhu W., Wang H., Huang J., Cheng L., Zhou H., Wu J., Tian Y. (2015). J. Mater. Chem. C.

[cit32] Hu R., Leung N., Tang B. (2014). Chem. Soc. Rev..

[cit33] Lu P., Lam J., Liu J., Jim C., Yuan W., Xie N., Zhong Y., Hu Q., Wong K., Cheuk K. (2010). Macromol. Rapid Commun..

[cit34] Qin A., Lam J., Tang B. (2012). Prog. Polym. Sci..

[cit35] Taniguchi R., Yamada T., Sada K., Kokado K. (2014). Macromolecules.

[cit36] Wu Y., Hu J., Huang H., Li J., Zhu Y., Tang B., Han J., Li L. (2014). J. Polym. Sci., Part B: Polym. Phys..

[cit37] Wang K., Yang J., Gong C., Lu H. (2017). Faraday Discuss..

[cit38] Wang K., Lu H., Liu B., Yang J. (2018). Eur. Polym. J..

[cit39] Schales O., Schales S. S. (1941). J. Biol. Chem..

[cit40] Lowe E. R., Banks C. E., Compton R. G. (2005). Electroanalysis.

[cit41] Cuartero M., Crespo G. A., Ghahraman Afshar M., Bakker E. (2014). Anal. Chem..

[cit42] Xue P., Ding J., Shen Y., Gao H., Zhao J. (2017). Dyes Pigm..

[cit43] Han T., Zhang Y., Feng X., Lin Z., Tong B., Shi J., Zhi J., Dong Y. (2013). Chem. Commun..

[cit44] Guo Y., Feng X., Han T., Wang S., Lin Z., Dong Y., Wang B. (2014). J. Am. Chem. Soc..

[cit45] Wu Y., Hu J., Huang H., Li J., Zhu Y., Tang B., Han J., Li L. (2014). J. Polym. Sci., Part B: Polym. Phys..

[cit46] Bineci M., Bağlan M., Atılgan S. (2016). Sens. Actuators, B.

[cit47] Li X., Wang C., Wan Y., Lai W., Zhao L., Yin M., Huang W. (2016). Chem. Commun..

[cit48] Li Y., Zhou H., Chen W., Sun G., Sun L., Su J. (2016). Tetrahedron.

