# Supplementary material for: Mechano-fluorochromic behavior of AEE polyurethane films and their high sensitivity to halogen acid gas
Source: RSC Adv. 2019 Mar 26;9(17):9517–21. doi: 10.1039/c8ra10486g (PMC9062156; doi:10.1039/c8ra10486g)
Supplement: RA-009-C8RA10486G-s001 [file RA-009-C8RA10486G-s001.pdf]

Electronic Supplementary Material (ESI) for RSC Advances.
This journal is © The Royal Society of Chemistry 2019

Mechano-fluorochromic behavior of AEE polyurethane films and their high sensitivity to halogen acid gas

Kun Wang, Meng Wang, Hao Lu, Beibei Liu, Mingming Huang, Jiping Yang[1]

Key Laboratory of Aerospace Advanced Materials and Performance, Ministry of Education, School of Materials Science and Engineering, Beihang University, Beijing 100191, China

Experimental Section

1 Materials

4-Ethynylbenzaldehyde was purchased from Sukailu Chemical Company. Palladium chloride ($PdCl_2$) and tetrakis(triphenylphosphine)palladium(0) ($Pd(PPh_3)_4$) were purchased from J&K Chemical Company. 3,3'-[Oxybis(2,1-ethanediyloxy)]bis-1-propanamine (DPA-DEG), poly(tetrahydrofuran) (Mn=1000, PTMG1000), cupric bromide ($CuBr_2$) and 4,4-diphenylmethane diisocyanate (MDI) were obtained from Aladdin Chemical Company. And the PTMG1000 was dried at 105 °C in vacuum for 2 h before used. 1,4-Butanediol (BDO), tetrahydrofuran (THF), N,N′-dimethyl formamide (DMF), methylene dichloride, petroleum ether and acetonitrile were purchased from Beijing Chemical Factory. THF was dried by anhydrous magnesium sulfate for two days, then refluxed with sodium in the presence of benzophenone and distilled for use after the solvent turned blue. DMF was reduced-pressure distilled after drying fully over calcium hydride (CaH_2). Toluene, methanol and potassium carbonate aqueous solution were degassed prior to use. All other reagents and solvents were used as commercially purchased without further purification.

2 Instrumentation

^{1}H-NMR spectra were obtained on a Bruker AV400 spectrometer using deuterateddimethyl sulfoxide (DMSO-d_6) as solvent at 25 °C. UV-vis adsorption spectra were measured on a TU1901 UV–vis spectrometer at room temperature. Fluorescence spectra were obtained using F-7000

[1] Corresponding author. Tel: 010-82338955.
E-mail: jyang@buaa.edu.cn.

fluorescence spectrophotometer. Molecular weight and molecular weight distribution of polymers were determined from a Waters 150C GPC system, using THF as the eluent at a flow rate of 1.0 mL/min and a set of monodisperse polystyrenes as calibration standards. Thermal transitions of the polyurethanes were investigated using a DSC-60 Plus system at a heating rate of 10 °C/min.

3 Synthesis

The synthesis of STMPU was depicted in the past work.[1] And it was also summarized here for convenience.

3.1 (1Z,3Z)-1,4-dibromo-1,4-dibenzaldehydebuta-1,3-diene (DBDABD-CHO)

4-Ethynylbenzaldehyde was used as raw material instead of phenylacetylene to simplify the process as illustrated in Scheme S1. 4-Ethynylbenzaldehyde (5.0 g, 0.38 mmol), $PdCl_2$ (0.34 g, 0.002 mmol), $CuBr_2$ (25.74 g, 0.16 mmol) were added to a three-necked flask. The mixed solvents of acetonitrile (3.84 mL) and methylbenzene (192 mL) were added and the mixture was stirred at room temperature until complete consumption of starting material as judged by thin layer chromatography. After the mixture was filtered and evaporated, the residue was purified by flash column chromatography using dichloromethane/petroleum ether (1:2) as eluent. DBDABD-CHO was obtained as yellow solid, yield 15%. ^1H NMR (400 MHz, DMSO-d_6, ppm): δ = 10.07 (s, 1H, CHO), 8.00 (d, 4H, Ar-H), 7.56 (s, 1H, C=C-H).

3.2 4,4'-((1Z,3Z)-1,4-diphenylbuta-1,3-diene-1,4-diyl)dibenzaldehyde (TABD-CHO)

DBDABD-CHO (0.4 g, 0.10 mmol), phenylboronic acid (0.27 g, 2.16 mmol), Pd(PPh3)4 (0.06 g, 0.04 mmol) and potassium carbonate (0.79 g, 5.72 mmol) were added to a three-necked flask. Under an argon atmosphere, the mixed solvents of toluene (60 mL) and methanol (20 mL) were added and the mixture was stirred and refluxed for 24 h. After the reaction was finished, the product was extracted with trichloromethane ($CHCl_3$) and washed with brine. The organic layer was combined and dried with $MgSO_4$. The crude product was purified by flash column chromatography using dichloromethane/petroleum ether (1:1) as eluent. TABD-CHO was obtained as yellow solid,

yield 85%. ^{1}H NMR (400 MHz, DMSO-d6, ppm): 9.95 (s, 1H, CHO), 7.83 (d, 2H, Ar-H), 7.54 (m, 3H, Ar-H), 7.33 (m, 4H, Ar-H), 6.80(s, 1H, C=C-H).

3.3 4,4'-((1Z,3Z)-1,4-diphenylbuta-1,3-diene-1,4-diyl)dibenzaldehyde (TABDAA2)

DPA-DEG (0.117 g, 0.51 mmol) was dissolved in 5 mL chloroform in a 50 mL round bottom flask. TABD-CHO (0.1 g, 0.23 mmol) and a few drops of glacial acetic acid were dissolved in 30 mL chloroform and added dropwise into the flask. The reaction mixture was stirred and refluxed for 24 h. After the reaction was finished, the resulting product was extracted with $CHCl_3$ and washed with brine for several times. When removing the solvent by reduced pressure distillation and drying at 50 °C for 48 h under vacuum, the product was obtained as brown yellow viscous liquid, yield 63%. ^{1}H NMR (400 MHz, DMSO-d_6, ppm): 8.10-7.63 (m, 8H, Ar-H), 7.55-7.11 (m, 21H, Ar-H), 6.66 (ddd, 2H, Ar-H), 4.62-4.29 (m, 2H, -CH), 3.63-3.10(m, 15H, $-CH_2$), 1.36-1.00(m, 15H, $-CH_3$).

3.4 STMPUs

MDI and anhydrous DMF were added to the flask with magnetic stirring under N2 atmosphere. TABDAA2 was dissolved in anhydrous DMF, added into the flask and reacted in room temperature for 0.5 h. Then PTMG1000 was dissolved in anhydrous DMF and added into the mixture solution in 60 ℃. After heating and stirring for 2 h, BDO was added dropwise and reacted for another 2 h. Finally, the mixture was poured into the water, the resulting precipitates were isolated by filtration after being washed with water and ethyl alcohol for several times, then put into vacuum oven until stable weight, yield ~95%.

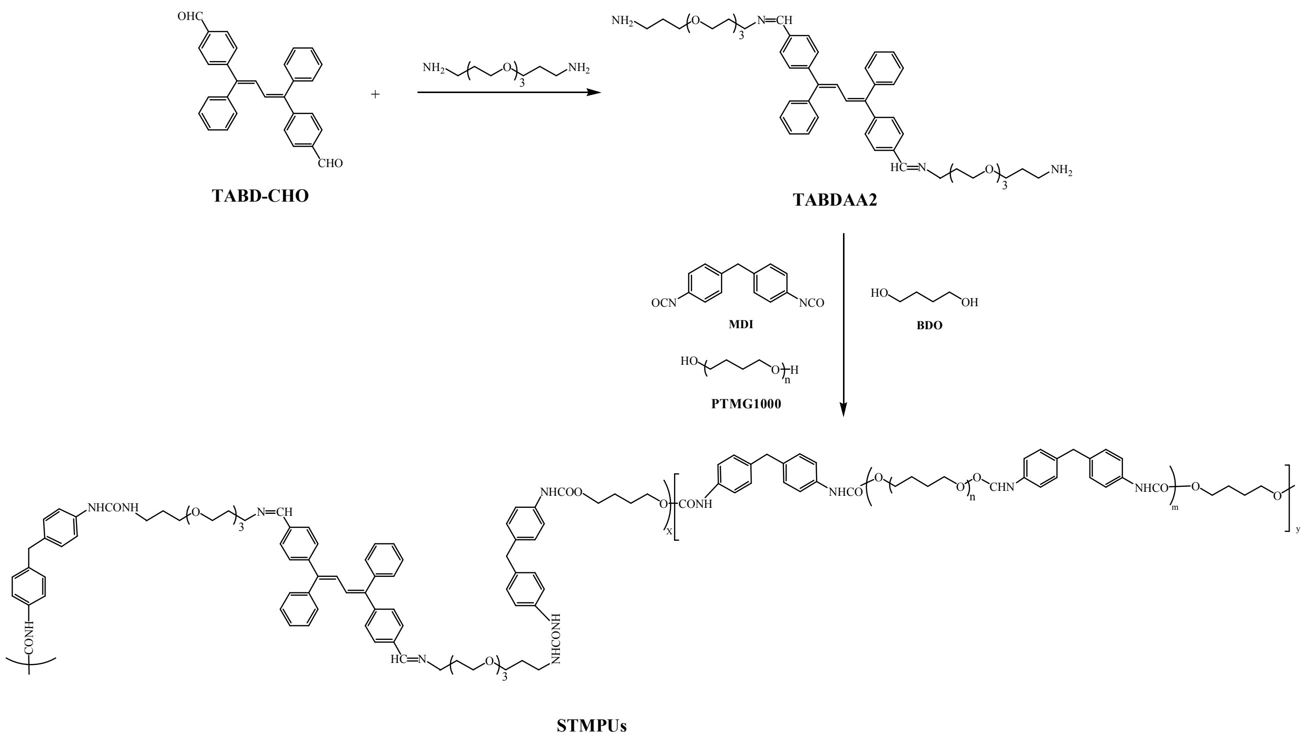

Scheme S1 Synthesis pathway of modified polyurethanes STMPUs.

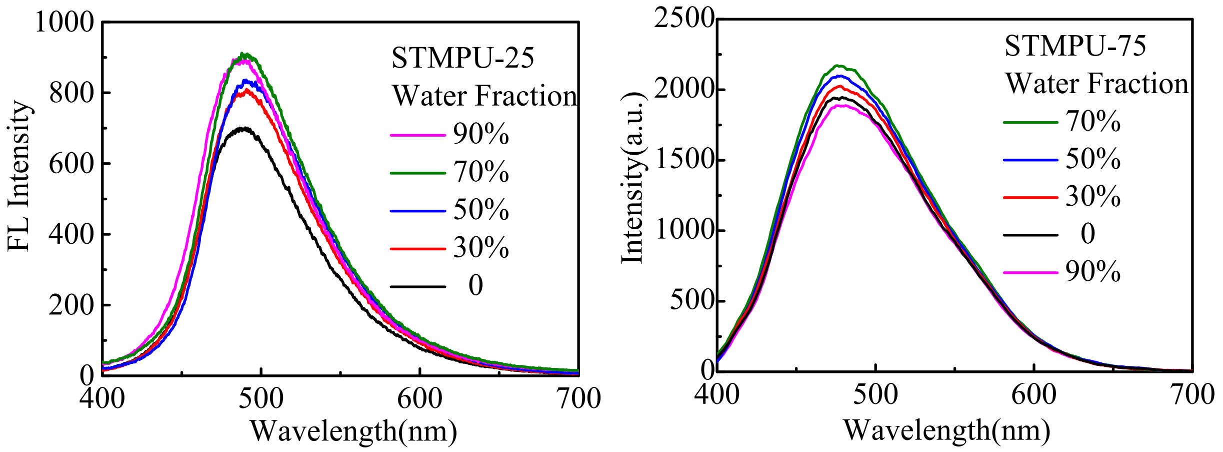

Figure S1 Fluorescence emission spectra of a) STMPU-25 and b) STMPU-75 in DMF–water mixtures with different water fractions (polyurethane solution concentration: 10 mg/mL; excitation wavelength: 365 nm).

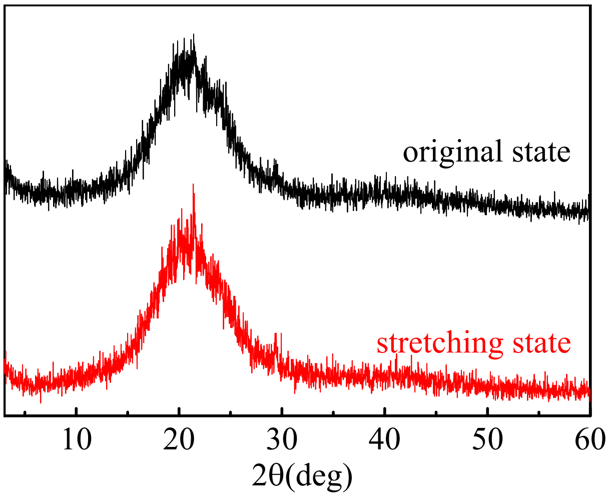

Figure S2 XRD profiles of STMPU-50 film before and after stretching (ε_T = 0.6).

Reference:

1. K. Wang, H. Lu, B. Liu and J. Yang, *Eur. Polym. J.*, 2018, **101**, 225-232.
